# Insect Receptors: Biochemical, Physiological and Molecular Studies

**DOI:** 10.3390/biom12050657

**Published:** 2022-04-30

**Authors:** Dov Borovsky

**Affiliations:** Department of Biochemistry and Molecular Genetics, University of Colorado Anschutz Medical School, Aurora, CO 80045, USA; dovborovsky@gmail.com

A *Biomolecules* Special Issue on insect receptors was a great opportunity to invite colleagues from all over the world to contribute original articles and timely reviews on the subject. Insect receptors control a myriad of important physiological events in the life of insects; thus, current and up-to-date reports on the function of these receptors provide new insight into their function, allowing researchers to glean new ideas on the physiological events that take place in insects with the hope that some of these physiological phenomena can be harnessed to control those insects that are agricultural pests or are transmitting diseases, such as mosquitoes.

**Borovsky et al.** [1] studied the binding of *Aea*TMOF to elucidate the mechanism by which TMOF stops the translation of the trypsin transcript in gut epithelial cells. These authors showed for the first time that *Aea*TMOF binds the ribosome at the exit trunnel, preventing the translation of the late trypsin mRNA. Using in vitro highly purified *Escherichia Coli* ribosome, the authors showed that *Aea*TMOF, a proline rich peptide, acts in a similar fashion to the known function of oncocin 112 that was shown by X-ray crystallography to bind at the entrance of the peptide exit tunnel of the *Thermos Thermophilus* ribosome, blocking the binding of formyl-tRNA to the A-site and, thus, stopping the translation of the mRNA and protein syntheses. Borovsky et al., using 3D modeling, showed that *Aea*TMOF also binds at the entrance of the peptide exit tunnel below the A-site of *T. Thermophilus*, *E. coli* and *Drosophila melanogaster* ribosome. They studied the kinetic of the binding of *Aea*TMOF to the *E. coli* ribosome, showing high affinity K_D_ = 23 ± 3.4 nM and B_max_ = 0.553 ± 0.023 pmol/μg ribosome. Using in vitro *E. coli* or *Spodoptera frugiperda* protein extracts containing 70S and 80S ribosomes, they showed that luciferase biosynthesis and larval late trypsin were inhibited in the presence of *Aea*TMOF at an IC_50_ of 1.0 μM and 1.8 μM, respectively. This study strongly suggests that the ultimate receptor target of *Aea*TMOF is the ribosome.

**Koutroumpa et al**. [2] studied sex pheromone receptors in *Spodoptera littoralis* and the development of the macroglomeruli in the antennal lobe where pheromone signals are processed. These authors used CRISPER-Cas9 to knock out the receptor for the major component of the sex pheromone to study the effect on the electrophysiological responses of peripheral pheromone-sensitive neurons. They showed that knocking out the receptor only affected antennal neurons, but it did not affect the number of macroglomeruli; however, it reduced the size of the macroglomerulus and the processing input from neurons that are tuned to the main pheromone component. They suggested that the *S. littoralis* mutant show, for the first time, that it lacks neuronal activity because of the absence of the pheromone receptor, which reduces the neural connectivity between peripheral and antennal lobe neurons.

**Tzotzos [3]** reviewed, compared and evaluated the structure and dynamic properties of insect odorant binding proteins. The author indicated that insects use a major part of their metabolic resources to produce odorant-binding proteins (OBP). The OBP role is not known, even though they initially were thought to be solubilized bind and transport semiochemicals to olfactory receptors. OBPs, nevertheless, play a diverse but not known role in insect physiology. The structures of the majority of the OBPs shed some light on their potential roles, but the dynamic properties of these proteins unfortunately have not received scrutiny. It is known, nevertheless, that native protein folding enabled OBPs to adapt to protein substrate binding. The author provides a comprehensive review of the structure and dynamic properties of OBPs using sequence structure analysis including physical and statistical approaches. The review adds new information and methodical tools to find a relationship between protein 3D structure and dynamics by using elastic network models and potential functions of OBPs in insects. Three-dimensional structures were used to study protein motions, which may shed light on ligand recognition, binding, and their conserved structural cores as related to evolutionary and functional importance.

**Montino et al. [4]** showed that, in *Tribolium castaneum* (Coleoptera, Tenebrionidae), two Odorant Binding Proteins (OBPs) that are encoded by gene pair *TcasOBP9A* and *TcasOBP9B* are close homologs of *Drosophila melanogaster* OBP Lush (*Dmel*OBP76a). These OBPs mediate pheromone reception in the insect. Electroantennographic analyses conducted by these authors show that the two *T. castaneum* OBPs are not pheromone specific, but their role is to enhance the detection of organic volatiles. Both OBPs are expressed in the antenna, and despite their homology and location on the chromosome, they have appeared at the base of Cucuijformia about 200 Mio years ago. The authors postulate based on their data that this gene pair is not the result of a gene duplication event that happened in different sensilla, but they are the result of being used as a co-OBP functional enhancer. 

**Schilcher et al. [5]** followed the important roles that octopamine and tyramine play in sensory responses in honeybees facilitating learning, memory and social organization. Therefore, Schilcher et al. studied the functions of tyramine and octopamine in honeybees in response to light using electroentinography to find the effects on the sensory sensitivity of the photoreceptor. The authors found that maximal receptor responses were detected using octopamine, whereas tyramine caused a decrease in receptor response. Phototaxis experiments to study behavioral responses relative to light after treatment with each amine showed that octopamine increased the walking speed of bees toward different light sources, whereas tyramine decreased walking speeds independent of locomotor activity. The authors concluded that their results show that tyramine and octopamine in honeybees cause opposite effects in response to light.

**Borovsky et al. [6]** cloned, sequenced and characterized the *Aedes aegypti* gut receptor of Trypsin Modulating Oostatic Factor (TMOF). Although TMOF has been sequenced and characterized 28 years ago, its gut receptor was difficult to solubilize, clone and characterize. Using a new approach, Borovsky et al. finally solubilized the TMOF receptor from the guts of female *Ae. aegypti* and isolated and purified the protein using affinity chromatography. The protein was separated on SDS-PAGE, and a protein sequence of 1306 amino acids was identified as ATP Cassette Binding protein (ABC) in the genome of *Ae. aegypti* by MS/MS analysis. To study the properties of the TMOF receptor, the receptor cDNA was cloned into plasmid pTAC-MAT-2, and *E. coli* cells were transformed to express the receptor in the inner bacterium membrane. Fluorescence-labeled TMOF with FITC was then used to characterize the binding to *E. coli* cells expressing the receptor on the inner bacterial membrane. TMOF exhibited high affinity binding to its receptor (K_D_ = 113.7 ± 18 nM ± SEM and B_max_ = 28.7 ± 1.8 pmol ± SEM). The incubation of TMOF-FITC with genetically modified bacterial cells that expressed ABC-TMOF receptor/importer showed that these bacterial cells bound the receptor and imported it into bacterial cells using fluorescence microscopy. Three-dimensional modeling of the receptor indicates that the receptor has ATP binding sites, and TMOF transported into the cells can be inhibited using ATPase inhibitors such as Arsenate or Na Azide. 

**Jackson and Gäde [7]** studied the interactions between the red pigment concentrating hormone (RPCH) of *Daphnia pulex* and its cognate receptor. These authors used single amino acids replacements using an Ala scan approach of Dappu-RPCH ligand and then docked the Ala-replaced ligand to its receptor. They proceeded to calculate the binding energies and compared them with the original, unaltered ligand binding energy. Several approaches were used, including molecular dynamics (MD) in the presence of palmitoyl-2-oleoyl-sn-glycero-3-phospahte (POPC). A good inverse correlation was observed for most Ala-changed Dappu-RPCH between in vitro activity and binding. However, it is interesting to note that [Ala4]Dappu-RPCH bound as tightly as the cognate ligand but showed little activity. MD data analysis showed that although [Ala4]Dappu-RPCH exhibited multiple interactions with the receptor, the interactions were different than the interaction that was exhibited by the natural Dappu-RPCH. Apparently, a strong binding affinity of the ligand to the receptor is not a prerequisite for activation. Binding to Ser155 and Gln237 is essential for activation. These observations in combination with published experimental results validate the Dappu-RPCH R model.

**Choi and Vander Meer** [8] reported a new novel system that is easy to use and can quickly screen randomly expressed short peptides using a phage display library. The authors indicated that many neuropeptides have their cognate G-protein coupled receptors (GPCR) and, thus, are ideal to be used as biological targets to control insects. They named their technique as receptor interference (RECEPTORi). The authors used genetically engineered Sf9 cells that expressed the fire ant’s GPCR on the cell membrane and a phage library expressing 10^9^ short peptides. After extensively washing the cells that were bound, phages that expressed short peptide(s) were identified, and the sequenced bound phages and peptides that were bound to the fire ants’s GPCR were identified. Several of the tightly bound peptides that were identified were injected into fire ants, and the biological activities of these peptides were determined. The authors claim that the identified peptides can be efficiently delivered by feeding insects with genetically engineered plants that express the peptides in a bait such as sugar or by topical applications. The author suggests that their report provides the first proof of concept for developing arthropod pest-management strategy using neuropeptide(s) in combination with GPCR.

**Holtof et al. [9]** studied the role of juvenile tolerant hormone-receptor Methoprene and Taiman during the sexual maturation of adult male locusts. These JH receptor components were shown to transduce JH signals in adult males. Therefore, these authors used dsRNA to knockdown these JH receptor components and reported that male maturation and reproduction were severely inhibited. The affected males did not exhibit mating behaviors, did not have yellow colored cuticle, showed a reduction in testes weight and their pheromone levels of phenylacetonitrite were also reduced. The dsRNA-treated males lost weight compared with the controls that were not treated with dsRNA. The organ that synthesizes JH, *corpora allata*, significantly increased in size and the transcript level of Juvenile hormone acid methyl transferase, a rate-limiting enzyme in the biosynthetic path of JH, increased. The authors reported that other endocrine pathways were also affected: the expression levels of insulin-related peptide and two neuroparsins in the locust fat body. These results indicate that the JH signaling pathway receptor components, such as Methoprene tolerant and Taiman, play an essential role in the male’s reproductive physiology and could be used in the future as targets for newly developed insecticides. 

**Toprak et al. [10]** reviewed intracellular calcium and its relationship to insect rynodine and Inositol 1,4,5-triphosphate receptors. The authors indicated that the endoplasmic reticulum (ER) is a major reservoir for Ca^2+^, whereas the inositol 1,4,5-triphosphate receptor (IP_3_R) and the rynodine receptor (RyR) are associated with the ER and actively played a role in providing the needed Ca^2+^ supply. Most information on these receptors was derived from mammalian systems that encode for three genes for each receptor, whereas the insect system on the other hand encodes for one gene for each receptor. The current review tries to answer the question of why there are three genes each in the mammalian system and one gene each in insect systems. The review covers the discovery of these genes in insects, examines their structure as related to their functions and the pathways that they interact with and their potential utility in future pest control. The authors show that RyR and IP_3_R receptors share structural similarities; however, they have been shown to be phylogenetically distinct and have their own structural organization, regulatory mechanisms and expression patterns that may explain why they are functionally different. The authors also reviewed the potential of these receptors for future pest control and point out that RyR is currently being targeted by a commercial insecticide named diamides.

**Rihani et al. [11]** tried to answer a 40-year-old mystery question: What is the physiological role(s) of insect odorant-binding proteins? Insect survival depends on their ability to detect molecules present in their environment; therefore, odorant-binding proteins (OBPs) are important for their survival and form a family that is involved in chemoreception. These proteins are found in olfactory appendages and in chemosensory and non-chemosensory organs. They have the ability to bind, solubilize and transport hydrophobic stimuli to chemoreceptors across the aqueous sensilla lymph. They can also mitigate sudden changes in odorant levels and are involved in hygro-receptions as well. The authors indicate that the role of the OBPs that are found in other body parts, such as mouthparts, pheromone glands, reproductive organs, digestive tract and venom gland, is not known, and they provide an up-to-date comprehensive overview of the roles of these OBPs in many tissues by examining their tissue expression levels and possible functional roles in insects.
